# Bcipep: A database of B-cell epitopes

**DOI:** 10.1186/1471-2164-6-79

**Published:** 2005-05-29

**Authors:** Sudipto Saha, Manoj Bhasin, Gajendra PS Raghava

**Affiliations:** 1 Institute of Microbial Technology Chandigarh, India

## Abstract

**Background:**

Bcipep is a database of experimentally determined linear B-cell epitopes of varying immunogenicity collected from literature and other publicly available databases.

**Results:**

The current version of Bcipep database contains 3031 entries that include 763 immunodominant, 1797 immunogenic and 471 null-immunogenic epitopes. It covers a wide range of pathogenic organisms like viruses, bacteria, protozoa, and fungi. The database provides a set of tools for the analysis and extraction of data that includes keyword search, peptide mapping and BLAST search. It also provides hyperlinks to various databases such as GenBank, PDB, SWISS-PROT and MHCBN.

**Conclusion:**

A comprehensive database of B-cell epitopes called Bcipep has been developed that covers information on epitopes from a wide range of pathogens. The Bcipep will be source of information for investigators involved in peptide-based vaccine design, disease diagnosis and research in allergy. It should also be a promising data source for the development and evaluation of methods for prediction of B-cell epitopes. The database is available at .

## Background

The antigenic regions of protein recognized by the binding sites of immunoglobulin molecules are called B-cell epitopes [1]. These epitopes can be classified into two categories; i) conformational/discontinuous epitope, where residues are distantly separated in the sequence and brought into physical proximity by protein folding and, ii) linear/continuous epitope, comprised of a single continuous stretch of amino acids within a protein sequence that can react with anti-protein antibodies [2,3]. Most of the B-cell epitopes were thought to be discontinuous. However, in late 1980s it was shown that this conformational restriction is not a necessary condition for the production of protein-reactive anti-peptide antibodies [4]. The designing of the conformational epitopes is difficult and so experimental B-cell epitopes largely include linear epitopes. These linear epitopes can be exploited in the development of synthetic vaccines and disease diagnosis. A number of vaccines based on B-cell epitopes are currently under clinical phase trials against viruses [5], bacteria [6] and cancer [7]. These epitopes are also important for allergy research and in determining cross-reactivity of IgE-type epitopes of allergens [8].

A large number of B-cell epitopes have been reported in the literature in last two decades. There is a need to collect and compile these epitopes to evaluate the performance of existing B-cell epitope prediction methods and to further develop better methods [9,10]. We have observed that the performance of the existing physico-chemical scales is not very high [11]. Recently, Blythe and Flower examined 484 amino acid propensity scales in predicting of B-cell epitopes and found that even the best set of scales and parameters performed only marginally better than random [12]. The evaluation of the existing scales indicates that there is a need to develop better methods by using artificial intelligence techniques. The collection of B-cell epitopes should help in deriving new scales for accurate in silico prediction of linear epitopes. Also it will help the immunologist to understand the complex nature of immunogenic peptides and for the development of vaccines. There are many databases available on T-cell epitopes [13-15]. In contrast, there are limited number of databases on B-cell epitope for example JenPep [16,17] and HIVDB [18]. Recently, JenPep has been superseded by AntiJen 2.0, which has included peptides bound to MHC ligand, TCR-MHC Complexes, T cell epitope, TAP, B cell epitope molecules and immunological protein-protein interactions. AntiJen also contains peptide library, copy numbers and diffusion coefficient data. Though AntiJen provides information about different types of peptides (>24000 entries) from a single source but it provides limited information about B-cell epitopes and tools to analyze and retrieve the data. Recently, we have created a comprehensive database, Bcipep, of B-cell epitopes. Latest version of Bcipep contains 3031 entries, where each entry provides detailed description of a B-cell epitope. Currently, we have covered only the continuous B-cell epitopes. The aim of this database is to assist the scientific community working in the areas of synthetic peptide vaccines (based on B-cell epitopes) and allergy research. The database will complement the existing databases such as AntiJen [16,17].

### Availability

The database is available at ,  (Mirror Site) and  (SRS version).

## Database construction

The PostgreSQL relational database management system (RDBMS) has been used for storing, retrieval and managing the data. The scripts, which provide interface between user and database, were written in PERL, CGIPerl and Pgperl. B-cell epitopes were collected from the literature (PubMed, ; ScienceDirect, ). A large number of HIV epitopes were extracted from a book [18].

## Database description

**Figure 1:**
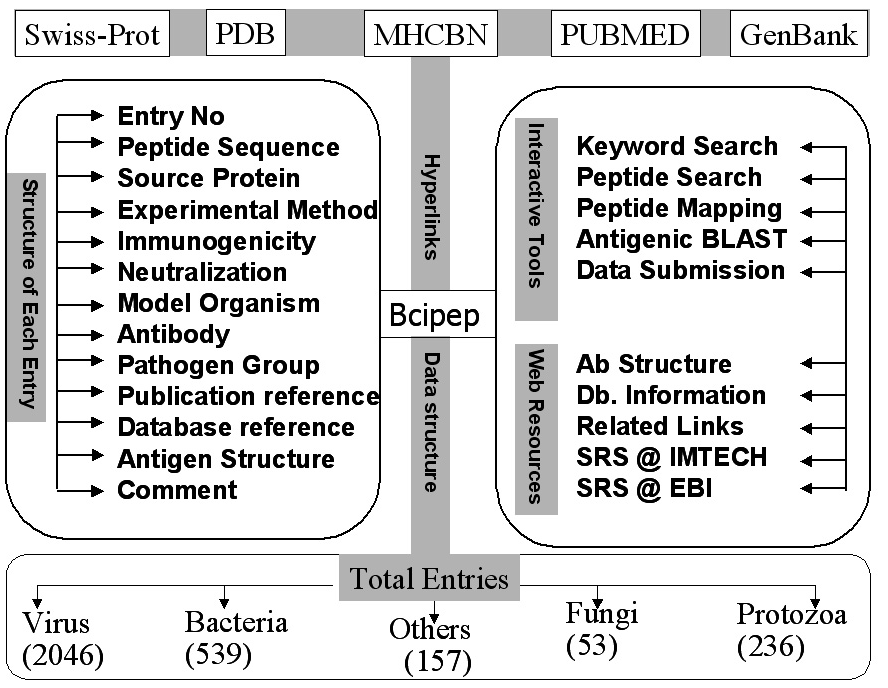
A schematic representation of Bcipep database.

The aim of Bcipep database is to provide; i) comprehensive information about B-cell epitopes, ii) tools for extraction and analysis of this information and, iii) hyperlinks to related databases. The overall architecture of Bcipep database is shown in Figure 1.

## Database information

### General

This database provides compehensive information about the linear B-cell epitopes which includes; i) amino acid sequence of epitope, ii) source protein from which epitopes were obtained, iii) experimental methods used in accessing the immunogenic potential of epitopes, iv) pathogen group (e.g., bacteria, virus, fungi, protozoa) of source protein and, v) miscellaneous information in the '*Comment' *field.

### Immunogenicity and model organisms

The immunogenicity of a peptide is a semi-quantitative measure of its immunogenic potency. In Bcipep it is divided into three categories; i) immunodominant, if it increases 2–3 folds anti-peptide antibodies in comparison to reference or control (carrier protein, eg., BSA or KLH), ii) immunogenic, if it enhances anti-peptide antibodies by one-fold in comparison to reference, and iii) null-immunonogenic, where no difference was observed when compared to reference. This information is very important for developing B-cell epitope prediction method. The database also provides information about '*Model organism' *used for immunization.

### Antibodies and neutralization

The database provides full information about monoclonal or polyclonal antibodies produced against an epitope. The information includes, isotypes of immunoglobin and name/number of monoclonal antibodies. The database also contains information about neutralization potential of anti-peptide antibody, which is crucial for considering a peptide for synthetic vaccine design.

### Links to databases

The Bcipep provides hyperlinks to various sequence databases in order to provide detailed information about peptides in database. The '*database reference*' field consists name/code of protein available in SWISS-PROT [19]. The '*Antigenic structure*' field consists of PDB codes [20] of protein structures having matching peptides. These PDB codes are linked to OCA browser  in order to provide detailed structure information of these proteins. It also provides structure information about 242 antibodies, where each antibody is hyperlinked to PDB database through the OCA browser. The '*Publication reference*' field provides full information about related publications with link to PUBMED [21]. Bcipep is also linked to MHCBN database [15] in order to identify the peptides that are B-cell as well as T-cell epitopes.

## Web tools

Bcipep has following major web-based tools for retrieval and analysis of information in Bcipep database (Figure 1). These web tools have been designed to facilitate the user in retrieving information from database.

### Keyword search

**Figure 2:**
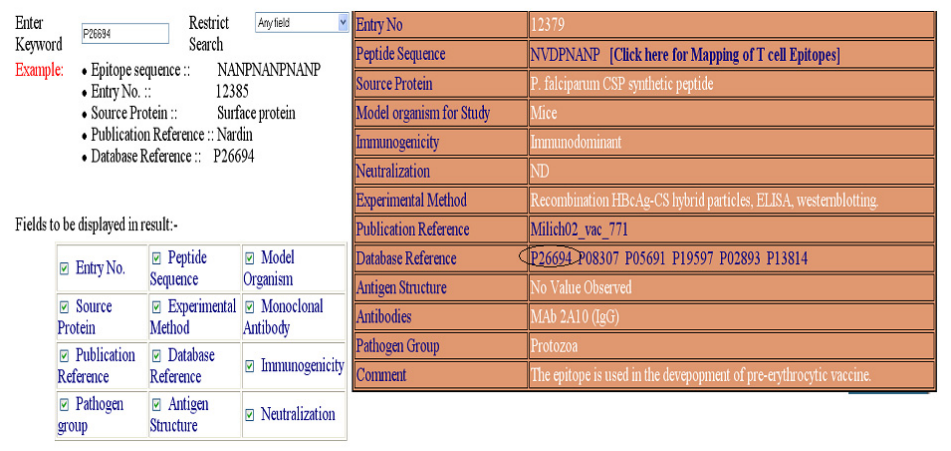
The typical display of Bcipep database for keyword search; a) input page of keyword search; and b) output of keyword search.

This option allows users to perform search on all fields of the database ('*Peptide Sequence', 'Source Protein', 'Publication Reference', 'Database Reference*'). One can restrict the keyword search on any specific field. It also allows users to select the fields to be displayed. An example of keyword search is shown in Figure 2a, where key word 'P26694' is searched in any filed of database. The output/result of this keyword search is shown in Figure 2b.

### Peptide search

**Figure 3:**
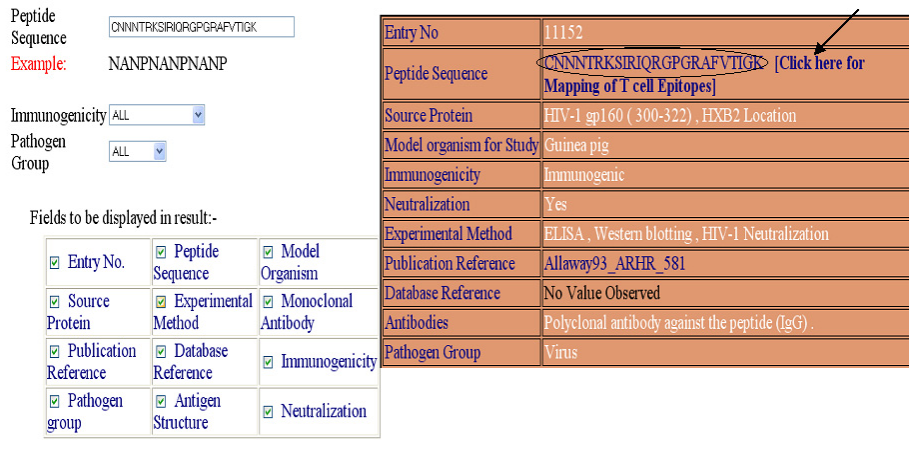
This example illustrate peptide search on Bcipep; a) Peptide search page; and b) result of peptide search.

The database provides option to search a peptide in Bcipep. The tool will display full information about the peptides included in Bcipep. The server also permits users to search their query sequence in any pathogen group. Search can be restricted on the basis of immunogencity that is immunodominant, immunogenic or null-immunogenic. An example of input and output of peptide search is shown in Figures 3a and 3b respectively.

### Mapping of T-cell epitopes

**Figure 4:**
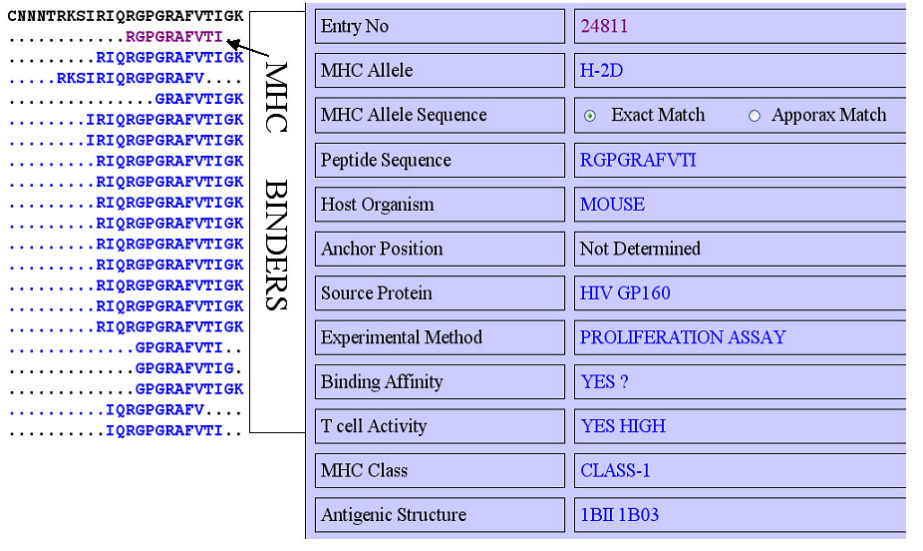
Mapping of peptide in MHCBN database; a) mapping of MHCBN peptides on B-cell epitope, and b) full information about a MHCBN peptide.

This server allows searching of peptide in Bcipep against MHCBN database. The MHCBN database provides information about components of cell-mediated immunity like MHC binders/non-binders, T-cell epitopes and TAP binders [14]. The peptides related to cell-mediated immunity can be mapped on resultant B-cell epitopes obtained from keyword/peptide search by clicking on '*Peptide Sequence' *field (Figures 2b and 3b). Thus, the server is useful in identifying the potential B-cell epitopes having T-cell epitopes (or MHC binders). The example of mapping of MHCBN peptides on B-cell epitope (by clicking on peptides sequence field of Figure 3b) is shown in Figure 4a. The full information of each map peptide can be obtained by clicking on the mapped sequence. One such example is shown in Figure 4a and 4b. The mapping allows user to detect the regions in B-cell epitope having promiscuous MHC binders (peptides that can bind to large number of MHC alleles) or T-cell epitopes.

### Peptide mapping

**Figure 5:**
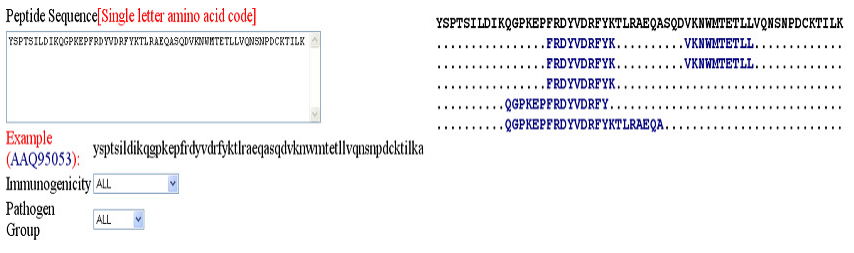
Mapping of B-cell epitopes on antigen sequence, a) submission page of B-cell epitope mapping, and b) mapping results

The peptides of Bcipep can be mapped on query sequence using this option. The full information about mapped peptide can be obtained by clicking on it. The tool will assist the researchers in gaining knowledge about the known immunogenic or non-immunogenic regions in target protein of interest. The example input and output of peptide mapping is shown in Figures 5a and 5b respectively. The users can specify the pathogen group and/or immunogenicity level of peptides to be mapped on query sequence. As shown in Figure 5b, the graphical mapping of peptides/epitopes on query allows one to easily detect the regions that bind to large number of B-cell peptides.

### BLAST search

This tool allows users to search their query protein against antigenic proteins maintained at Bcipep. The sequence of 1070 antigenic proteins has been obtained from SWISS-PROT. The similarity search is performed using the GWBLAST server . The GWBLAST also allows users to analyze the BLAST output like multiple alignments, phylogenetic analysis.

## Online data submission

The database has the facility to submit data to Bcipep on-line via Internet. Users can submit information about their experimentally determined B-cell epitopes. We hope that immunologists will submit their information on B-cell epitope in Bcipep, similar to the sequence data at GenBank and SWISS-PROT. The Bcipep team will add more data from literature to maintain B-cell data up-to-date and cross check the data submitted by users to maintain quality of the data.

## Potential utility and limitations

One of the major challenges in the field of subunit vaccine design is to identify the antigenic regions (B and T cell epitopes) that can generate antigen specific memory cells. Thus, the identification of regions/stretches on an antigen from the data pool of known epitopes is an important step in vaccine design. The Bcipep database would be very useful as it consists of comprehensive information about experimentally verified linear B-cell epitopes and tools for mapping these epitopes on an antigen sequence. In case query antigen contains known epitopes, this database might aid in the wet experimentation and lower the cost by reducing the overlapping repeats. This strategy is frequently used for the screening of transgenic proteins by searching linear IgE-binding epitopes [22]. Unlike discontinuous epitopes, the linear epitopes are easy to design, as they do not require tertiary structure information. Bcipep also provides information on neutralizing B-cell epitopes where an antibody generated against a B-cell epitope neutralizes the parent antigen. The current version of Bcipep provides neutralizing information on about 1309 such B-cell epitopes. This information is very important for selecting functional B-cell epitopes. This database also provides a link with MHCBN to search for overlapping regions of MHC binders and T-cell epitopes in the B-cell epitopes. Thus, the user can identify both antigenic regions that can activate B-cell and T-cell, which can lead to the development of better vaccine. The epitopes in Bcipep can be used to derive the rules for predicting B-cell epitopes.

The aim of designing synthetic linear peptides as epitope-vaccine is to induce neutralizing antibodies against the pathogen [23]. There are many reports that the linear B-cell epitopes were characterized as neutralizing antibodies as in Clostridium botulinum neurotoxin type A (Btx A)[24]. In Bcipep, there are 748 neutralizing anti-peptide antibodies entries. However, in some cases these linear epitope(s) fail to produce neutralizing antibodies and do not give protective immunity. For instance, it has been shown in the past that the antibodies against the synthetic peptides and short recombinant proteins of approximately 100 amino acids of hepatitis E virus (HEV) do not neutralize, suggesting that the HEV neutralization epitope(s) is conformation dependent [25]. The elicitation of a bactericidal and protective immune response to *Borrelia burgdoferi *decorin binding protein requires a properly folded conformation for the production of functional antibodies [26]. Recently, Corcoran *et al*, 2004, observed that B-cell memory is established and maintained against conformational epitopes of Parvovirus VP2 and against linear epitopes of VP1 but not against linear epitope VP2 [27]. Thus, it is not necessary that the linear B-cell epitope will always give rise to memory cells. One should also check the neutralizing information of B-epitopes, as only 748 B-cell epitopes out of 1309 in Bcipep were able to neutralize the parent protein.

For an effective use of Bcipep, it is important to understand the limitation of linear B-cell epitopes and data in Bcipep. The few limitations of current version of Bcipep are; i) it does not cover discontinuous epitopes, ii) it has limited number of unique peptides (1590) in 3031 entries and, iii) it contains peptides having only natural amino acids. One should be careful in using linear B-cell epitopes in developing epitope based subunit vaccine. The organism used for immunization (information included in the database) should also be taken into consideration, since immune response is T-helper cell (MHC-II-peptide complex) dependant and B-cell epitope alone may not generate protective antibodies [28]. In some cases, the nature of the adjuvant used and the route of immunization (information not included in the database) might also play important roles in the induction of protective anti-peptide antibody response against the pathogen [29-31].

## Authors' contributions

SS collected and compiled the data as well as developed the web server. MB helped in designing website and stored data in PostgreSQL. GPSR conceived the idea and supervised the work.

## References

[B1] Van Regenmortel MH (1993). Synthetic peptides versus natural antigens in immunoassaya. Ann Biol Clin (Paris).

[B2] Barlow DJ, Edwards MS, Thornton JM (1986). Continuous and discontinuous protein antigenic determinants. Nature.

[B3] Langeveld JP, martinez-Torrecuadrada J, boshuizen RS, Meloen RH, Ignacio CJ (2001). Characterisation of a protective linear B cell epitope against feline parvoviruses. Vaccine.

[B4] Walter G (1986). Production and use of antibodies against synthetic peptides. J Immunol Methods.

[B5] El Kasmi KC, Muller CP (2001). New strategies for closing the gap of measles susceptibility in infants: towards vaccines compatible with current vaccination schedules. Vaccine.

[B6] Sabhanini L, Manocha M, Sridevi K, Shashikiran D, Rayanade R, Rao DN (2003). Developing subunit immunogens using B and T cell epitopes and their constructs derived from F1 antigen of *Yersinia pestis *using novel delivery vehicles. FEMS Immunol Med Microbiol.

[B7] Kieber-Emmons T, Luo P, Qiu J, Chang TY, Insung O, Blaszczyk-Thurin M, Steplewski Z (1999). Vaccination with carbohydrate peptide mimotopes promotes anti-tumor responses. Nat Biotechnol.

[B8] Selo I, Clement G, Bernard H, Chatel J, Creminon C, Peltre G, Wal J (1999). Allergy to bovine beta-lactoglobulin:specificity of human IgE to tryptic peptides. Clin Exp Allergy.

[B9] Odorico M, Pellequer JL BEPITOPE: predicting the location of continuous epitope and patterns in proteins. J Mol Recognit.

[B10] Alix AJ (1999). Predictive estimation of protein linear epitopes by using the program PEOPLE. Vaccine.

[B11] Saha S, Raghava GPS, Nicosia G, Cutello V, Bentley PJ, Timmis J (2004). BcePred: Prediction of continuous B-cell epitopes in antigenic sequences using physico-chemical properties. ICARIS, LNCS.

[B12] Blythe MJ, Flower DR (2005). Benchmarking B cell epitope prediction: Underperformance of existing methods. Protein Science.

[B13] Brusic V, Rudy G, Harrison LC (1998). MHCPEP, a database of MHC-binding peptides: update 1997. Nucleic Acids Res.

[B14] Rammensee H, Bachmann J, Emmerich NP, Bachor OA, Stevanovic S (1999). SYFPEITHI: database for MHC ligands and Peptide Motifs. Immunogenetics.

[B15] Bhasin M, Singh H, Raghava GPS (2003). MHCBN: A comprehensive database of MHC binding and non-binding peptides. Bioinformatics.

[B16] Blythe MJ, Doytchinova IA, Flower DR (2002). JenPep: a database of quantitative functional peptide data for immunology. Bioinformatics.

[B17] McSparron H, Blythe MJ, Zygouri C, Doytchinova IA, Flower DR (2003). JenPep: a novel computational information resource for immunobiology and vaccinology. J Chem Inf Comput Sci.

[B18] Korber B, Brander C, Haynes B, Koup R, Kuiken C, Moore J, Walker B, Watkins D (2002). HIV Monoclonal Antibodies. "HIV Molecular Immunology 2001".

[B19] Bairoch A, Apweiler R (2000). The SWISS-PROT protein sequence database and its supplement TrEMBL in 2000. Nucleic Acids Res.

[B20] Westbrook J, Feng Z, Jain S, Bhat TN, Thanki N, Ravichandran V, Gilliland GL, Bluhm WF, Weissig H, Greer DS, Bourne PE, Berman HM (2002). The Protein Data Bank: unifying the archive. Nucleic Acids Res.

[B21] Wheller DL, Church DM, Lash AE, Leipe DD, Madden TL, Pontius JU, Schuler GD, Schriml LM, Tatusova TA, Wagner L, Rapp BA (2002). Database resources of National Center for Biotechnology Information: 2002 update. Nucleic Acids Res.

[B22] Kleter GA, Peijnenburg AA (2002). Screening of transgenic proteins expressed in transgenic food crops for the presence of short amino acid sequences identical to potential, IgE – binding linear epitopes of allergens. BMC Structural Biology.

[B23] Xiao Y, Lu Y, Chen YH (2001). Epitope-vaccine as a new strategy against HIV-1 mutation. Immunol Lett.

[B24] Wu HC, Yeh CT, Huang YL, Tarn LJ, Lung CC (2001). Characterization of neutralizing antibodies and identification of neutralizing epitope mimics on the Clostridium botulinum neurotoxin type A. Appl Environ Microbiol.

[B25] Meng J, Dai X, Chang JC, Lopareva E, Pillot J, Fields HA, Khudyakov YE (2001). Identification and characterization of the neutralization epitope(s) of the hepatitis E virus. Virology.

[B26] Ulbrandt ND, Cassatt DR, Patel NK, Roberts WC, Bachy CM, Fazenbaker CA, Hanson MS (2001). Conformational nature of the Borrelia burgdorferi decorin binding protein A epitopes that elicit protective antibodies. Infect Immun.

[B27] Corcoran A, Mahon BP, Doyle S (2004). B cell memory is directed toward conformational epitopes of parvovirus B19 capsid proteins and the unique region of VP1. J Infect Dis.

[B28] An LL, Whitton JL (1997). A multivalent minigene vaccine, containing B-cell, cytotoxic T-lymphocyte, and Th epitopes from several microbes, induces appropriate responses in vivo and confers protection against more than one pathogen. J Virol.

[B29] Obeid OE, Stanley CM, Steward MW (1996). Immunological analysis of the protective responses to the chimeric synthetic peptide representing T- and B-cell epitopes from the fusion protein of measles virus. Virus Res.

[B30] Fernandez IM, Snijders A, Benaissa-Trouw BJ, Harmsen M, Snippe H, Kraaijeveld CA (1993). Influence of epitope polarity and adjuvants on the immunogenicity and efficacy of a synthetic peptide vaccine against Semliki Forest virus. J Virol.

[B31] Todryk SM, Kelly CG, Lehner T (1998). Effect of route of immunisation and adjuvant on T and B cell epitope recognition within a streptococcal antigen. Vaccine.

